# Supplementary material for: SARS-CoV-2 main protease inhibition through dimerization promotion by peptidomimetic inhibitors and disruption by ebselen
Source: eLife. 2026 Oct 26;15:RP109956. doi: 10.7554/eLife.109956 (PMC13707381; doi:10.7554/eLife.109956)
Supplement: Peer review history

# Editor report: eLife Assessment

**DOI:** 10.7554/eLife.109956.4.sa0

**Authors:** Chang Liu

**Affiliations:** Johns Hopkins University United States

**Keywords:** Convincing, Important

This **important** study compares how different classes of drugs act on the SARS-CoV-2 main protease, a key antiviral target, and shows that many of them work by controlling whether the enzyme assembles into its active dimeric form. The evidence, based on a range of complementary biophysical methods, is **convincing** and points to the interface between the two protein protomers, including a newly found binding site, as a promising target for broad-spectrum antiviral drugs. This work will be of interest to biochemists and virologists working on treatments for coronaviruses.

# Referee report: Reviewer #2 (Public review)

**DOI:** 10.7554/eLife.109956.4.sa1

Summary:

This manuscript presents a sophisticated investigation into the mechanisms by which different inhibitor classes affect the SARS-CoV-2 main protease (Mpro), a pivotal antiviral drug target. This study reveals that effective inhibition can be achieved by modulating the stabilization of the essential dimeric state. It also indicates the dimer interface could be a druggable allosteric site, which may offer a strategy for developing broad-spectrum anticoronaviral agents.

Strengths:

The identification of dimer interface stabilization/destabilization as distinct inhibitory mechanisms and the discovery of C300 as a potential allosteric site for ebselen are important contributions to the field. The experimental approach is modern, multi-faceted, and generally well-executed.

Comments on latest version:

All of my concerns have been adequately addressed.

# Author comment: Author response

**DOI:** 10.7554/eLife.109956.4.sa2

**Authors:** Chengxi Liu, Qinyu Jia, Chang Zhao, Yao Zhongping

**Affiliations:** Hong Kong Polytechnic University Hong Kong Hong Kong; Hong Kong Polytechnic University Hong Kong Hong Kong; Hong Kong Polytechnic University Hong Kong Hong Kong; Hong Kong Polytechnic University Hong Kong China

The following is the authors’ response to the previous reviews.

**Reviewer #1 (Public review):**

Summary:

Since dimerization is essential for SARS-CoV-2 Mpro enzymatic activity, the authors investigated how different classes of inhibitors, including peptidomimetic inhibitors (PF-07321332, PF-00835231, GC376, boceprevir), non-peptidomimetic inhibitors (carmofur, ebselen, and its analog MR6-31-2), and allosteric inhibitors (AT7519 and pelitinib), influence the Mpro monomer-dimer equilibrium using native mass spectrometry. Further analyses with isotope labeling, HDX-MS, and MD simulations examined subunit exchange and conformational dynamics. Distinct inhibitory mechanisms were identified: peptidomimetic inhibitors stabilized dimerization and suppressed subunit exchange and structural flexibility, whereas ebselen covalently bound to a newly identified site at C300, disrupting dimerization and increasing conformational dynamics. This study provides detailed mechanistic evidence of how Mpro inhibitors modulate dimerization and structural dynamics. The newly identified covalently binding site C300 represents novelty as a druggable allosteric hotspot.

Strengths:

This manuscript investigates how different classes of inhibitors modulate SARS-CoV-2 main protease dimerization and structural dynamics, and identifies a newly observed covalent binding site for ebselen.

Weaknesses:

None. The requested mutagenesis data have been provided in the revised manuscript, and all of my previous concerns have been satisfactorily addressed.

**Recommendations for the authors:**

**Reviewer #1 (Recommendations for the authors):**

None. The overall quality of the manuscript has been substantially improved. The authors have added supportive mutagenesis data in the revised manuscript to validate the proposed role of C300. All of my concerns have been adequately addressed.

We appreciate the reviewer’s recognition of the improvements made in the revised manuscript.

**Reviewer #2 (Public review):**

Summary:

This manuscript presents a sophisticated investigation into the mechanisms by which different inhibitor classes affect the SARS-CoV-2 main protease (Mpro), a pivotal antiviral drug target. This study reveals that effective inhibition can be achieved by modulating the stabilization of the essential dimeric state. It also indicates the dimer interface could be a druggable allosteric site, which may offer a strategy for developing broad-spectrum anticoronaviral agents.

Strengths:

The identification of dimer interface stabilization/destabilization as distinct inhibitory mechanisms and the discovery of C300 as a potential allosteric site for ebselen are important contributions to the field. The experimental approach is modern, multi-faceted, and generally well-executed.

Comments on revised version:

The authors have very nicely addressed most of the previous comments raised. But one comment remains to be clarified relating to original point 5 and the authors' response:

"We agree with the reviewer about the need for quantitative rigor in reporting HDX changes. We have calculated the fractional deuterium uptake difference for each peptide fragment discussed in the text between the inhibitor-bound and unbound states. These values, along with their statistical significance (p-values from a two-tailed t-test), have been provided in the revised manuscript (Legends for Figures 3 and 4). Although the HDX change of residues 296-306 is relatively small (<5%), this region showed a reproducible difference with low experimental variability and statistical significance (p < 0.05). Given its location within the C-terminal dimerization interface and its consistency with native MS, we interpret this change as a subtle local conformational perturbation."

Two questions remain for the statements in line 376-380. First, while it is stated "residues 296-304 in the C-terminal region of Mpro were more flexible upon ebselen binding", the segment of 296-306 is shown Figure 4c. Second, the HDX change for this segment upon ebselen binding is very subtle in the figure (in contrast to the significant HDX change of the same segment in the protein upon PF-07321332 binding), thus making the strong conclusion that "This suggests that ebselen targeting C300 may induce structural changes in the C-terminal helical segment, weakening key hydrogen bonds at the dimer interface and ultimately inhibiting activity" not convincing. The reviewer would suggest the authors either delete this conclusion or largely tone it down.

We thank the reviewer for the recognition of our efforts and agree with the reviewer’s suggestion. We have corrected “residues 296–304” to “residues 296–306” in Line 377 and removed the statement “This suggests that ebselen targeting C300 may induce structural changes in the C-terminal helical segment, weakening key hydrogen bonds at the dimer interface and ultimately inhibiting activity.”, as suggested.
